# Actin-Binding LIM 1 (ABLIM1) Inhibits Glioblastoma Progression and Serves as a Novel Prognostic Biomarker

**DOI:** 10.1155/2022/9516808

**Published:** 2022-12-20

**Authors:** Danping Liu, Xiaoping Wang, Ying Liu, Chunliu Li, Zhen Zhang, Peng Lv

**Affiliations:** ^1^Department of Neurology, Yantai Yuhuangding Hospital, Yantai, Shandong 264000, China; ^2^Department of Clinical Laboratory, Yantai Affiliated Hospital of Binzhou Medical University, Yantai, Shandong 264100, China; ^3^Department of Neurology, Yantai Affiliated Hospital of Binzhou Medical University, Yantai, Shandong 264100, China; ^4^Department of Quality Control Division, Yantai Yuhuangding Hospital, Yantai, Shandong 264000, China

## Abstract

**Background:**

Glioma is the most prevalent malignant brain tumor in adult humans, and glioblastoma (GBM) is the most malignant type. The actin-binding LIM 1 (ABLIM1) protein can modulate actin polymerization, which is essential for the cell proliferation and migration. We aim to investigate ABLIM1 expression, function, and clinical significance in GBM.

**Methods:**

The *ABLIM1* mRNA level was extracted from the TCGA and GTEx online databases. The ABLIM1 protein expression level was explored using immunohistochemistry staining in a GBM cohort enrolled in our hospital (*n* = 104). The patient survival and prognostic factors were determined using the Kaplan-Meier method and multivariate Cox hazard proportional analysis, respectively. Two human GBM cell lines, U87 and U251 cells, were utilized for ABLIM1 overexpression and cell proliferation analyses. A subcutaneous xenograft model was generated using nude mice to validate the tumor-related effect of ABLIM1 *in vivo*.

**Results:**

*ABLIM1* exhibited a significantly lower mRNA level in GBM than in other glioma or normal brain tissues. Higher ABLIM1 protein level was correlated with smaller GBM tumor size and better cancer-specific survival (CSS). Multivariate analysis identified ABLIM1 as a novel independent prognostic factor for GBM prognosis. ABLIM1 overexpression significantly inhibits U87 and U251 cell proliferation and colony formation. Consistently, ABLIM1 exerted tumor-suppressing functions in mice models.

**Conclusion:**

ABLIM1 plays antitumor roles in GBM progression and could be served as a novel biomarker to help predict GBM prognosis.

## 1. Introduction

Glioma is the most prevalent malignant brain tumor in adult humans. According to the World Health Organization (WHO), glioblastoma (GBM) is classified as grade IV glioma, highlighting its highly aggressive phenotype. The current treatment for GBM is surgical resection followed by radiochemotherapy [1]. Considering most GBM patients suffer from self-care difficulty, careful hospital nursing is critical for better life quality and prognosis. The Karnofsky score is a significant prognostic factor for GBM; the GBM prognosis is unsatisfactory. The five-year survival time of GBM is 5.5%, with a median survival time of less than 15 months [2, 3]. The intrinsic molecular characteristic of GBM in different patients is the predominant factor that results in distinct outcomes [4]. Therefore, GBM-related biomarker identification and their functional mechanism elucidation are urgently needed to improve disease prognosis.

The LIM domain refers to a cysteine-rich sequence motif that functions as a protein-binding interface and mediates specific protein-protein interactions. The actin-binding LIM (ABLIM) protein family members consist of four amino-terminal LIM domains and a carboxyl-terminal dematin-like domain, including a villin headpiece (VHP) domain that binds to actin filaments [5]. Currently, three subtypes, ABLIM1, ABLIM2, and ABLIM3, have been reported in mammals [6]. Actin-binding LIM 1 (ABLIM1) is a novel protein identified by Roof et al. in 1997 [7]. ABLIM1 can bind F-actin and thus mediates interactions between actin and other proteins. ABLIMs can synergistically stimulate the striated muscle activator of Rho signaling- (STARS-) dependent activation of the serum-response factor (SRF) through binding tightly with F-actin [8].

Dysregulated ABLIM1 is correlated with various diseases. ABLIM1 can construct actin networks to prevent mechanical tension-induced blebbing [9]. *ABLIM*1 abnormal splicing is identified in skeletal muscles of myotonic dystrophy type 1 patients and the corresponding mouse model [10]. The ablation of a retina-specific isoform of ABLIM1 results in the absence of morphological and functional defects in retinal neurons [11]. Similarly, *ABLIM1*-rs7099208 is associated with severe spermatogenic failure [12]. *ABLIM1* polymorphisms are also associated with personality traits and alcohol dependence [13], implying its significant role as an axon development-related protein [14]. An in silico study suggests that hypermethylated and lowly expressed ABLIM1 is present in gestational diabetes mellitus [15]. Meanwhile, ABLIM1 participates in osteoclast differentiation and migration, depending on the NF-*κ*B ligand (RANKL)-mediated signaling pathway [6, 16].

*ABLIM1* maps to human chromosome 10q25, a frequently deleted region in human cancers [17]. According to microarray data, ABLIM1-mRNA is downregulated in adrenocortical carcinomas compared to nontumorous tissues [18]. The tumor-related role of ABLIM1 has also been reported in hepatocellular [19] and nasopharyngeal carcinoma [20]. However, whether ABLIM1 modulates GBM progression is unknown. Here, we aim to investigate the ABLIM1 expression profile in GBM for the first time. Additionally, correlations between ABLIM1 with patients' clinicopathological characteristics and survival will be analyzed. Finally, functional mechanisms of ABLIM1 in GBM will be explored using *in vitro* and *in vivo* assays.

## 2. Methods

### 2.1. Online Dataset

The clinical information and mRNA gene expression data were obtained from the TCGA (https://cancergenome.nih.gov/) and GTEx (https://gtexportal.org/home/) databases. The survival information was retrieved from Liu et al.'s publication [21].

### 2.2. Cohort Enrollment

GBM tissues were collected from Yantai Yuhuangding Hospital (*n* = 104). Patients with pathologically confirmed non-GBM and patients with incomplete clinical features were excluded. The study was conducted with all participants' informed consent and approved by the Ethics Committee of Yantai Yuhuangding Hospital (No. 2022-280).

### 2.3. Immunohistochemistry (IHC)

ABLIM1 protein expression was detected in GBM tissues using IHC. Briefly, the sections were heated at 60°C overnight, dewaxed with water, and heated for antigen retrieval. Then, sections were incubated with 1% H_2_O_2_ to inactivate endogenous enzymes for 10 min at room temperature. The sections were incubated with anti-ABLIM1 primary antibody (1 : 50, #MBS2518798, MyBioSource, USA) overnight in the cold room, then with the secondary antibody for 30 min at room temperature the following day. The 3,3′-diaminobenzidine (DAB) was used for color staining, followed by hematoxylin incubation for 5 min. The stained IHC sections were observed under a light microscope (400x magnification). The staining was assessed based on staining intensity and percentage of positively stained cells by two independent pathologists blinded to patients' information. Sections with greater than 50% positively stained cells (dark yellow or brown) were considered high-ABLIM1 expression. Otherwise, sections were considered low-ABLIM1 expressions.

### 2.4. Cell Culture and Transfection

Two human GBM cell lines, U87 (wild-type *TP*53) and U251 (mutant *TP*53), were used in our study. Both cell lines were maintained in DMEM supplied with 10% fetal bovine serum (FBS) and 1% penicillin/streptomycin. All cells were cultured at 37°C in a humidified atmosphere containing 5% CO_2_. Human *ABLIM*1 cDNA was cloned into pcDNA3.1 plasmids for overexpressing transfection serving as a negative control, according to the manufacturer's instructions.

### 2.5. Western Blot

After lysing cultured cells with radioimmunoprecipitation assay (RIPA) buffer (Beyotime, China), equal amounts of proteins were separated by sodium dodecyl-sulfate polyacrylamide gel electrophoresis (SDS-PAGE) and transferred to polyvinylidene difluoride (PVDF) membranes (Millipore, USA). Then, transferred membranes were blocked with 5% BSA and incubated for 1 h at 4°C. The blocked PVDF membranes were incubated with primary antibodies, including anti-ABLIM1 (1 : 1000, #MBS2518798, MyBioSource, USA) and anti-beta-actin (1 : 3000; Proteintech, USA) antibodies, at 4°C overnight. On the next day, membranes were washed three times and incubated with the secondary antibody at room temperature for 1 h. The immunoblotting signals were visualized with an enhanced chemiluminescence (ECL) reagent (Thermo Fisher Scientific, USA).

### 2.6. Cell-Counting Kit 8 (CCK-8) Assays

The CCK-8 kit was used to evaluate cell proliferation activity according to the manufacturer's procedure. Briefly, 1,000 transfected cells were seeded into each well of a 96-well plate. Then, the cell proliferation capacity was measured at 0, 1, 2, 3, and 4 days. At designated time points, the culture medium was removed, and the cells were washed with phosphate-buffered saline (PBS) to remove dead cells. Then, CCK-8 reagents were added to the 96-well plates (100 *μ*L/well) and incubated at 37°C for 90 min. Finally, the absorption was measured at 450 nm using a microplate reader.

### 2.7. Colony Formation

Transfected cells were seeded into six-well plates 200 cells/well density cell and cultured for two weeks. Then, the colonies were fixed with methanol for 10 min, followed by staining with crystal violet for 20 min. The number of colonies was counted under a light microscope.

### 2.8. Mice Model

Four-week-old nude mice were injected subcutaneously with transfected cells to establish a xenograft model and examine the *in vivo* effect of ABLIM1 on GBM progression. The tumor size was monitored every week using the Vernier caliper. The tumor volume was calculated using the following formula: *V* (mm^3^) = (*L* × *W*^2^)/2 (*L* = tumor length and *W* = tumor width). All mice were sacrificed after four weeks. The xenografts were isolated and weighted. The study was approved by the Ethics Committee of Yantai Yuhuangding Hospital.

### 2.9. Statistics

Statistical analyses were conducted using SPSS (Version 20.0) and GraphPad Prism (Version 5.0) software. The correlations between ABLIM1 and clinic-pathological variables were tested using the chi-square test. Cancer-specific survival (CSS) was defined as the interval between the surgery and tumor-related death or the last follow-up. Univariate log-rank test and multivariate Cox regression model were used for survival analyses. Statistical differences between the two groups were assessed using Student's *t*-test. Independent experiments were repeated at least three times for each experiment and exhibited as mean ± standard deviation (SD). *P* < 0.05 was considered statistically significant [22].

## 3. Results

### 3.1. *ABLIM1* Expression and Clinical Relevance on mRNA Level Using Online Databases

**Figure 1:**
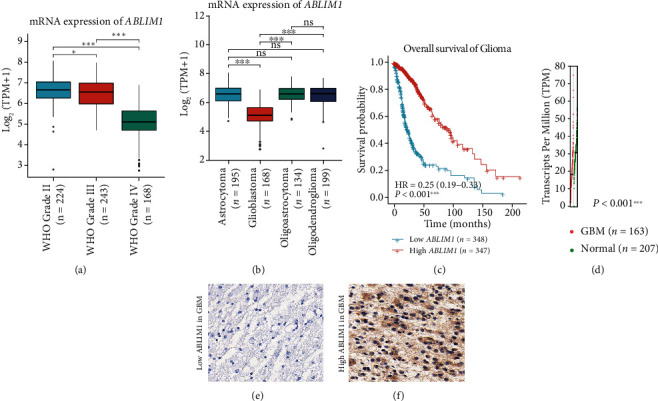
The *ABLIM1* mRNA and protein expression in GBM. (a) The *ABLIM1* mRNA level in glioma tissues with different WHO grades from the TCGA database was compared, indicating the lowest level in WHO grade IV glioma, namely the glioblastoma (GBM). The *y*-axis was plotted according to the transcripts per million (TPM) and converted to log_2_ (TPM+1). (b) The *ABLIM1* mRNA level in glioma tissues with different histological types from the TCGA database was compared, showing the lowest level in glioblastoma compared with other histological types. (c) We conducted a Kaplan-Meier survival analysis to compare the survival difference of glioma patients with low or high *ABLIM1* mRNA levels using the data from the TCGA database. (d) The *ABLIM1* mRNA level was compared between GBM and normal brain tissues using TCGA and GTEx databases (*P* < 0.001). Each red dot indicates a specific mRNA level in a GBM sample, while each green dot indicates normal brain tissue. (e) Representative low *ABLIM1* protein expression in GBM as reflected using IHC, demonstrating almost negative expression in specific GBM samples. (f) Representative high *ABLIM1* protein expression in GBM tissues using IHC, exhibiting a positive expression in the cytoplasm. ^∗^*P* < 0.05, ^∗∗∗^*P* < 0.001; ns: no significance. Magnification: 400x.

We discovered that *ABLIM1* exhibits distinct levels in glioma tissues with different WHO grades by extracting the microarray data from the TCGA database (Figure 1(a)). GBM tissues (WHO grade IV) had significantly lower *ABLIM1* levels than low-grade gliomas (WHO grade II-III). *ABLIM1* level comparison based on histological types revealed that GBM has the lowest *ABLIM1* level than astrocytoma or oligodendroglioma (Figure 1(b), *P* < 0.001). Notably, glioma patients with a lower *ABLIM1* level had shorter overall survival than those with a higher *ABLIM1* level (Figure 1(c), *P* < 0.001). We investigated *ABLIM1*'s detailed role in GBM due to its significantly lower level than other glioma types. As expected, TCGA and GTEx datasets revealed a significantly lower *ABLIM1*-mRNA level in GBM than in normal brain tissues (Figure 1(d)).

### 3.2. Enrolled Patients' Characteristics

Table 1 displays that the median age of our enrolled cohort (*n* = 104) is 62 years old. The GBM cohort included 51 females and 53 males. The median tumor size was 4.0 cm, recorded by the largest diameter. Among the 104 cases, 55 underwent local resection, 20 underwent radical resection, and the remaining 29 underwent lobectomy. Only 19 cases received definite chemotherapy, while the other 85 did not accept chemotherapy or were unsure. The median follow-up time was seven months, ranging from 1 to 132 months.

### 3.3. Protein Expression and Clinical Significance of ABLIM1 in GBM

**Figure 2:**
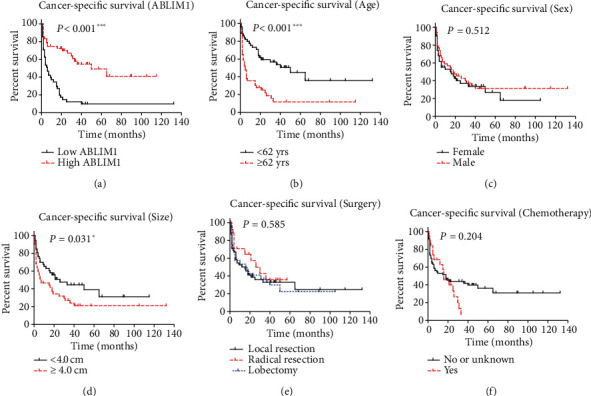
Cancer-specific survival (CSS) of GBM patients. CSS of the enrolled GBM cohort (*n* = 104) was analyzed using the Kaplan-Meier method according to patients' *ABLIM1* level (a), age (b), sex (c), tumor size (d), surgery (e), and chemotherapy (f). ^∗∗∗^*P* < 0.001 by log-rank test.

According to IHC (Figures 2(a) and 2(b)), different GBM tissues exhibited remarkable ABLIM1 protein expression levels. Accordingly, we divided enrolled GBM patients into low-ABLIM1 (*n* = 56) and high-ABLIM1 (*n* = 48) groups. The correlations between ABLIM1 and GBM characteristics were analyzed using the chi-square test (Table 1). ABLIM1 level was negatively correlated with tumor size (*P* = 0.029), indicating that ABLIM1 may exert tumor-suppressive effects in GBM. However, ABLIM1 presented no significant correlation between patient age, sex, surgery, and chemotherapy (*P* > 0.05).

The Kaplan-Meier method and log-rank test were conducted to assess patient prognosis (Table 2). Survival curves indicated that a lower ABLIM1 was correlated with a worse prognosis (Figure 2(a)), consistent with its prognostic significance of mRNA level (Figure 1(c)). The three-year CSS rate was 54.7% in the high-ABLIM1 group, while it decreased to 12.4% in the low-ABLIM1 group. Meanwhile, elderly patients had a poor prognosis (Figure 2(b), *P* < 0.001), highlighting the importance of careful nursing, while sex has no significant effect on patient survival (Figure 2(c), *P* = 0.512). Larger tumor size was significantly correlated with unfavorable survival as a conventional prognostic factor (Figure 2(d), *P* = 0.031). Nevertheless, neither the resection pattern nor chemotherapy had a prognostic effect in our cohort (Figures 2(e)–2(f), *P* > 0.05).

We applied patients' age, tumor size, and ABLIM1 level into a Cox hazard regression model to identify independent prognostic factors (Table 2). Consequently, elder age was an independent unfavorable prognostic factor (HR = 2.7, 95% CI 1.6–4.6, *P* < 0.001). Higher ABLIM1 protein expression was also determined as an independent favorable prognostic factor (HR = 0.3, 95% CI 0.2–0.6, *P* < 0.001), indicating that patients with higher ABLIM1 expression have better survival.

### 3.4. ABLIM1 Exerts an Anti-Proliferation Effect in GBM *In Vitro* and *In Vivo*

**Figure 3:**
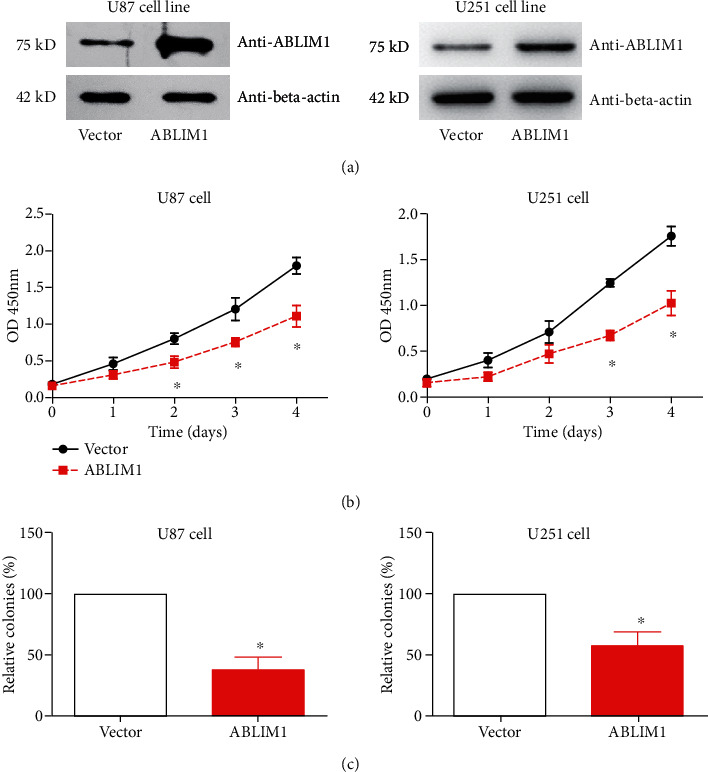
*ABLIM1* expression and function in GBM cell lines. (a) *ABLIM1* protein expression was tested after *ABLIM1*-plasmid transfection with vector plasmid as a control in U87 and U251 GBM cell lines using western blotting. (b) The CCK-8 assay was used to assess cell proliferation in transfected U87 and U251 cell lines. (c) The colony formation capacities of transfected cells were compared in transfected U87 and U251 cell lines. ^∗^*P* < 0.05 by Student's *t*-test.

The cellular experiments were conducted to evaluate the detailed function of ABLIM1 in GBM. Immunoblotting revealed that ABLIM1 was detectable in U87 and U251 cell lines. The transfection of ABLIM1 plasmids significantly increased its protein level (Figure 3(a)). The CCK-8 proliferation assay reflected an attenuated proliferation capacity in the ABLIM1-overexpressed group (Figure 3(b)). Consistently, ABLIM1 transfection impaired the colony formation ability in both cell lines (Figure 3(c)).

**Figure 4:**
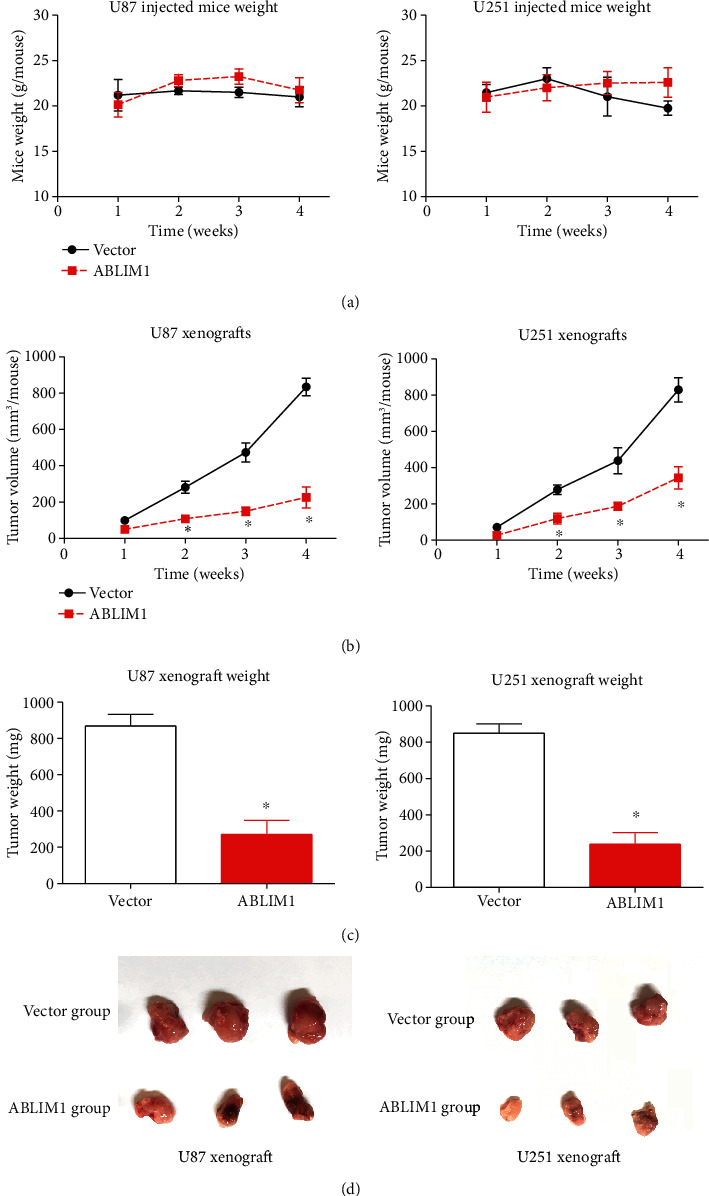
High *ABLIM1* suppressed GBM growth *in vivo*. (a) The mice weight was recorded since the date of subcutaneous tumor cell injection, showing no statistically significant difference between vector and *ABLIM1* groups. (b) Subcutaneous tumor growth was monitored weekly after tumor cell injection according to the tumor volume. The tumor volume was calculated using the formula: *V* (mm^3^) = (*L* × *W*^2^)/2 (*L* = tumor length and *W* = tumor width). (c) Four weeks after tumor cell injection, the xenografts were isolated and weighted to compare their differences. (d) Isolated xenografts were photographed, and visual size differences can be observed between vector and *ABLIM1* groups. ^∗^*P* < 0.05 by Student's *t*-test.

Finally, we established a subcutaneous xenograft model as an *in vivo* strategy to validate the role of ABLIM1 during GBM progression. Subcutaneous injection of different GBM cells had no significant effect on mice weight (Figure 4(a)). However, xenografts generated by ABLIM1-overexpressed cells exhibit decreased growth compared to those generated by vector-overexpressed cells (Figure 4(b)). After tumor excision and weighting, ABLIM1 xenografts were lighter and smaller than vector xenografts (Figures 4(c) and 4(d)). Therefore, we concluded that ABLIM1 might play antitumor roles by inhibiting GBM growth.

## 4. Discussion

Initially, we determined ABLIM1 expression and clinical significance in GBM. According to our data, decreased ABLIM1 was associated with larger tumor size, whereas ABLIM1 overexpression resulted in impaired GBM growth. Besides its total protein expression level, ABLIM1 phosphorylation has been previously reported to participate in hepatocellular carcinoma (HCC) progression. According to Dong et al.'s data, dominant negative mutations of Ser 214 and Ser 431 residues of ABLIM1 inhibited actin polymerization and affected cellular migration [19]. However, we cannot map the ABLIM1 phosphorylation level in GBM specimens due to lack of specific phosphorylation antibodies. Considering the importance of posttranslational modifications [23], whether its phosphorylation is dysregulated in tumors and the corresponding mechanism deserves further investigation.

Several studies revealed upstream regulators of ABLIM1. The lncRNA ZNF667-AS1 promoted ABLIM1 expression by adsorbing miR-1290, subsequently attenuating nasopharyngeal carcinoma progression [20]. Meanwhile, microarray data implied that miR-129-3p potentially targeted ABLIM1 in retinal pigment epithelial cells, thus inhibiting ciliation in proliferating cells and impairing cilia elongation [24]. The hsa-miR-6165 is another upstream microRNA of ABLIM1 in NT2 neural cells. The hsa-miR-6165 overexpression can downregulate ABLIM1, attenuate NT2 differentiation, and affect the cell cycle and apoptosis [25].

Nevertheless, we did not identify a statistically significant alteration in ABLIM1 expression after transfecting the above microRNAs in GBM cells (data not shown). The cell-type-specific signatures of microRNAs may explain this on target mRNA expression [26]. Additionally, ABLIM1 may interact with GSK-3*β* signaling pathways. A recent study demonstrated that GSK-3*β* could bind ABLIM1 and modulate its function in cardiovascular cells [27]. Since the Wnt-GSK-3*β* signaling pathway is well known for its tumor-related function, GSK-3 and ABLIM1 probably interact with malignancies. The upstream and downstream mechanisms of ABLIM1 in tumors must be systematically elucidated using high-throughput techniques, such as next-generation sequencing and mass spectrometry.

According to our clinical data, ABLIM1 expression in GBM tissues could be an independent prognostic factor. GBM patients with low or negative ABLIM1 expression have worse CSS. The important point is that elder age patients had a worse prognosis. Therefore, high-quality nursing is critical for GBM prognosis. However, our data were retrospectively collected from a single medical center with a limited case number, which may have a regional bias. Although we used an online database to verify our findings, further validation may be necessary. Nevertheless, we provided initial evidence regarding the tumor-related role of ABLIM1 in GBM from clinical, cellular, and *in vivo* aspects.

## 5. Conclusions

ABLIM1 is decreased in GBM, and its lower expression is correlated with poor prognosis. High ABLIM1 expression can inhibit GBM growth *in vitro* and *in vivo*.

## Figures and Tables

**Table 1:** Characteristics of GBM patients and their correlations with *ABLIM1* expression.

Variables	Cases	*ABLIM1* protein expression		*P* value
	(*n* = 104)	Low (*n* = 56)	High (*n* = 48)	
Age				
<62 yrs	51	23	28	0.079
≥62 yrs	53	33	20	
Sex				
Female	51	30	21	0.318
Male	53	26	27	
Tumor size				
<4.0 cm	53	23	30	0.029^∗^
≥4.0 cm	51	33	18	
Resection pattern				
Local resection	55	32	23	0.503
Radical resection	20	11	9	
Lobectomy	29	13	16	
Chemotherapy				
No or unknown	85	47	38	0.531
Yes	19	9	10	

^∗^ *P* < 0.05 by chi-square test.

**Table 2:** Cancer-specific survival analyses of GBM patients.

Variables	Cases	Univariate analysis		Multivariate analysis	
	(*n* = 104)	3-Y CSS rate	*P* value	HR (95% CI)	*P* value
Age					
<62 yrs	51	53.7%		Reference	
≥62 yrs	53	11.8%	<0.001^∗∗∗^	2.7 (1.6-4.6)	<0.001^∗∗∗^
Sex					
Female	51	33.6%			
Male	53	34.4%	0.512		
Tumor size					
<4.0 cm	53	44.5%		Reference	
≥4.0 cm	51	24.0%	0.031^∗^	1.1 (0.6-1.8)	0.812
Resection pattern					
Local resection	55	32.9%			
Radical resection	20	35.9%			
Lobectomy	29	35.9%	0.585		
Chemotherapy					
No or unknown	85	41.8%			
Yes	19	6.7%	0.204		
*ABLIM1* level					
Low	56	12.4%		Reference	
High	48	54.7%	<0.001^∗∗∗^	0.3 (0.2-0.6)	<0.001^∗∗∗^

^∗^ *P* < 0.05 by log-rank test. ^∗∗∗^*P* < 0.001. Abbreviations: *ABLIM1*; actin-binding LIM protein 1; CI: confidence interval; CSS: cancer-specific survival; GBM: glioblastoma; HR: hazard ratio.

## Data Availability

Data is available upon requirement.
